# Drone-based observations of scarring patterns in humpback whales (*Megaptera novaeangliae*) in the New York Bight provide insight into foraging behavior and anthropogenic threats

**DOI:** 10.1371/journal.pone.0324121

**Published:** 2025-05-30

**Authors:** Siobhan E. Keeling, Chelsi Napoli, Joshua Meza-Fidalgo, Julia S. Stepanuk, Nathan Hirtle, Zachary Hoffman, Lesley H. Thorne

**Affiliations:** 1 School of Marine and Atmospheric Sciences, Stony Brook University, Stony Brook, New York, United States of America; 2 New York State Department of Environmental Conservation, Division of Marine Resources. Kings Park, New York, United States of America; 3 Department of Ecology and Evolution, Stony Brook University, Stony Brook, New York, United States of America; 4 Maine Department of Marine Resources, West Boothbay Harbor, Maine, United States of America; MARE – Marine and Environmental Sciences Centre, PORTUGAL

## Abstract

Large whales face a range of threats, including vessel strikes and entanglement in fishing gear. Elevated humpback whale mortality, predominantly in juveniles, has occurred in the Northeast US since 2016. The New York Bight, a region with dense shipping and fishing vessel traffic, has become a hotspot for these strandings. Scarring patterns can provide information on anthropogenic threats, as well as predation and behavior. We used drone imagery to examine scarring reflective of entanglements, vessel strikes, killer whale interactions and bottom feeding in both juvenile and adult humpback whales in the New York Bight. The vast majority of both adult (87.1%) and juvenile (86.8%) humpbacks showed entanglement scars, indicating that humpbacks frequently interact with fishing gear across age classes. Vessel strike scars were observed more frequently in juvenile whales (14.2%) than in adults (2.2%), in contrast to prior observations north of our study area in the Gulf of Maine, though the difference was of borderline significance (mean *p*-value 0.051, Fisher’s Exact tests on 1000 bootstrapped populations incorporating uncertainty in length measurements). These results support previous suggestions that juvenile humpbacks in the New York Bight may be particularly vulnerable to vessel strike due to inshore and surface feeding, and suggest that vessel strike scars may be obtained locally. Killer whales are thought to primarily target young animals, and killer whale scars were observed more often in juveniles (11.6%) than adults (4.4%), though this difference was not significant (mean *p*-value 0.26). Jaw scuffing indicative of bottom feeding was observed more frequently in adults (68.9%) than in juveniles (27.4%; mean *p*-value 3.47 x 10^−5^), suggesting that this behavior is acquired as whales mature. Our findings underscore differences in behavior between adult and juvenile humpback whales and highlight the exposure of humpback whales to anthropogenic threats in heavily urbanized coastal regions.

## Introduction

Commercial whaling dramatically reduced large whale populations during the 20^th^ century [1,2]. While some populations of large whales have increased considerably since the International Whaling Commission’s moratorium of whaling was enacted in 1986, others have yet to recover [3–5]. Today, large whales face a range of threats, particularly vessel strikes and entanglement in fishing gear [6–10]. Scarring patterns can provide information on anthropogenic threats [11–13], predation [11,14,15] as well as behavior [16,17]. Assessing scars can provide a useful monitoring tool for large whales.

Entanglement in fishing gear can occur when foraging whales swim through lines or fishing gear suspended in the water column [18]. Entanglement in underwater gear can lead to drowning when the entangled whale is prevented from reaching the surface, or can lead to chronic entanglements in which the whale carries the entangled fishing gear for long periods of time [7]. As a chronically entangled whale swims through the water, the gear can cause deep lacerations that can become infected causing extreme discomfort and injury, including fluke or flipper amputation [19,20], or death [7,21]. Fishing gear types that are frequently involved in large whale entanglements include monofilament lines, buoy and ground lines from traps and pots, and netting from pot and gill fisheries [18,22–24]. The risk of drowning due to entanglement increases when there are multiple body regions that are entangled. Whales with five points of entanglement are more likely to succumb to drowning deaths than those with fewer points of entanglement [21]. Entanglements can leave scars that can be useful for identifying individuals that have been impacted by this threat. Entanglement scars can occur across the body but most frequently occur in areas such as the mouth, flippers, and caudal peduncle where the gear creates notch and wrap like scars [18,21,25–28]. Gear wrapping around the body or appendages can create scarring from tightening around a region or sliding back and forth [25,29].

Vessel strikes from a vessel’s hull or propeller can cause injury or mortality in large whales [30]. Vessel speed affects both the risk that a vessel strike will occur, as well as the severity of the injuries a whale will sustain when it is struck [30–32]. Faster vessel speeds increase the probability that a vessel strike will be fatal [31,33–35]. Whales are vulnerable to vessel strike while at the surface, and behaviors that increase the time spent at or near the surface can increase the risk of vessel strike [36–40]. Scars can be used to assess when individual whales have been struck by a vessel. Vessel strikes can cause sharp trauma, often resulting from contact with the propeller, which leaves distinctive scarring from the rotating blades of the vessel’s screw [41]. Vessel strike scars are typically more common on the animal's dorsal flank [30].

In addition to scars resulting from anthropogenic threats, scars resulting from interactions with predators or interactions with bottom substrate can provide information on large whale behavior and rates of predator attacks [15–17,42,43]. For example, humpback whales foraging on Stellwagen Bank in the Northeast US feed on sand lance along the bottom, which can produce abrasions on the lateral lower and upper jaw, or “jaw scuffing”, as the whales make contact with the bottom while foraging [16,17,44]. Lateralization has been evident in the foraging behavior of large whales [16,45,46], and scarring on the rostrum produced during bottom feeding in humpback whales can indicate which side that the individual favors to rotate onto while foraging [16,47]. In North Atlantic right whales, documented interactions with the seafloor increase the risk of entanglement in fishing gear [48], and feeding on the bottom may similarly make humpback whales more vulnerable to entanglement in bottom-set fishing gear [44]. Teeth marks from killer whales can be identified as multiple parallel lines called rake marks [49]. Since killer whale attacks are rarely observed directly, these scars can be used to assess the frequency of non-fatal interactions [15]. Killer whale predation can influence humpback whale habitat use; for example, killer whale predation is thought to be a factor causing humpback mothers with calves to preferentially use nearshore waters during migrations [50]. Assessing the occurrence of killer whale scars or scars reflecting bottom feeding can thus provide additional information or context for humpback whale habitat use, which can inform exposure to anthropogenic threats.

Until recently, studies of scars on larger whales primarily relied on boat-based photo-ID and observations [27,28,51]. Boat-based studies cannot typically determine scarring across the whole body and have largely focused on particular body regions such as the flukes [12,14,27,49,52] or the rostrum in studies of lateralization [16,17,47]. Unoccupied aerial systems (UAS) are now widely used in cetacean studies and are advantageous in that they can provide full-body aerial images [53–56]. UAS images can allow scarring to be assessed across the whole body, as opposed to specific body regions that are visible during boat-based photo-ID studies [12]. Whole-body images also allow multiple types of scarring to be examined concurrently, enabling analyses to examine the co-occurrence of different scar types.

In the Northeast United States, there is an ongoing unusual mortality event (UME) for humpback whales (NOAA 2022), and vessel strikes are thought to be an important driver of the increased mortality in this region [57]. During the UME, strandings of humpback whales have been particularly high in the New York Bight [57], a region of high vessel traffic and anthropogenic activity that provides foraging habitat for humpback whales during summer and fall [58–61]. Humpback whales foraging in New York waters are predominantly juveniles [61], which may be more susceptible to anthropogenic threats [62]. Assessing scarring in UAS images of humpback whales in the New York Bight in juvenile and adult whales would provide insight into the foraging behavior, drivers of habitat use, and threats facing humpback whales of different age classes and factors influencing the UME.

Our objectives herein were to: [1] Assess the frequency of entanglement, vessel strike, killer whale and jaw scarring on humpback whales in the New York Bight; [2] Investigate which and how many body regions showed scarring; [3] Compare the occurrence of scars between juveniles and adult humpback whales; and [4] Assess whether individuals showing evidence of bottom feeding showed higher rates of entanglement scarring.

## Methods

### Data collection

**Fig 1:**
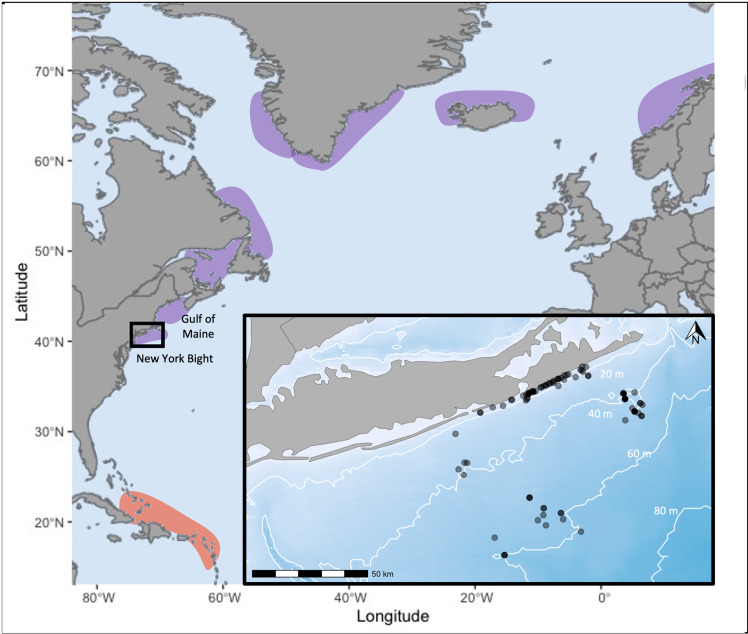
Location of the study area relative to breeding grounds (red shading) and broader foraging grounds (purple shading) of the West Indies Distinct Population Segment of Humpback whales. The study area is shown in the black rectangle is highlighted in the inset. The inset shows sampling locations of UAS imagery within the New York Bight. Figure made using Natural Earth Data https://www.naturalearthdata.com/.

UAS footage of humpback whales was obtained during opportunistic vessel-based surveys conducted in the New York Bight between May and November from 2018 to 2024 (Fig 1). To obtain UAS footage, a DJI Phantom 4 Pro + UAS (2018–2022) or a DJI Mavic 3 Pro UAS (2023–2024) was flown at an altitude between 15 and 40m. To help reduce surface disturbances, UAS flights were performed in conditions below 4 on the Beaufort scale. This work was conducted under a National Marine Fisheries Service General Authorization (GA No. 21889) from 2018–2021, and Permit No. 26260 from 2022–2024. UAS were flown by licensed Federal Aviation Administration (FAA) Part 107 pilots. The survey protocol for this work was approved by the Stony Brook University Institutional Animal Care and Use Committee.

Boat-based photographs of each humpback whale’s flukes and/or dorsal fins were used to photo-identify individual whales [63] and to identify whales that were resighted. For whales that were observed on more than one field day, whether within or between years, the best image(s) for that individual was selected for further analyses.

### Image processing

Still images were obtained from UAS video footage. Whenever possible, single full body images, in which the entire dorsal surface of the whale is visible at the surface, were taken from video footage. If a single full-body image of the whale at the surface was not attainable (e.g., flukes bent downward so the peduncle was not visible), separate still images were taken of multiple regions of the body during the same UAS flight. Additionally, video footage was examined to confirm that scarring patterns observed in stills were present on the whale’s body while in motion, and were not a misinterpretation of glare or rippling water on still images. Images were graded based on camera focus, angle and light as in Table 1, modified from [64]. If any attribute for an individual image received a score of 3 (poor), the image was excluded from analysis. Individuals were excluded from analysis if sufficient imagery (scores of 1 (good) or 2 (medium)) were not available for all regions of the body (Table 1).

**Table 1:** Criteria used to assess UAS images of humpback whales. Images were given a score of 1-3 for each attribute (camera focus, angle, light) and images scores of 1 (good) and/or 2 (medium) were included in analyses.

Attribute	Score 1 (good)	Score 2 (medium)	Score 3 (poor)
Camera Focus	The picture is sharp with unique patterns on the whale skin (e.g., scars, pigmentation) in clear focus	The picture is slightly blurry, but clear enough to distinguish unique patterns on the whale skin	The picture is too blurry to distinguish any unique patterns on the whale skin
Angle	The blowhole of the whale is aligned with the midline body axis of the whale, such that both sides of the dorsal surface of the whale are equally visible	The blowhole of the whale deviates slightly (<1/3 of the eye width) from the midline body axis of the animal, such that one side of the dorsal surface of the whale is slightly more visible than the other	The blowhole of the whale deviates significantly (>1/3 of the eye width) from the midline body axis
Light	Images are well lit	Glare or contrast slightly affect the visibility of pigmentation or scarring	Glare or contrast significantly affect the visibility of pigmentation or scarring

Each individual’s age class was determined based on measurements of total body length estimated from UAS photogrammetry. One of two methods were used to obtain accurate altitude measurements of UAS flights [65], which is key to accurately scaling morphometric measurements from UAS images. For data obtained in 2018, we used the method of Burnett et al. (2019), and used CollatriX [66] to correct UAS altitude estimates derived from the UAS barometer using measurements of an object of known size (the swim platform on our research vessel) taken at different heights. For data obtained thereafter, we used data from a SF11/c LiDAR laser altimeter installed on the UAS as in Dawson et al. (2017). The angle between the gimbaled camera and altimeter were accounted for as in Dawson et al. (2017). We measured total body length as in Stepanuk et al. (2021). Briefly, we conducted length measurements in MorphoMetriX (Torres and Bierlich 2020), with post hoc processing in CollatriX. MorphoMetriX uses the ground sampling distance equation to convert pixel measurements to cm [53,67]. Juvenile and adult whales were classified based on a threshold length of 11.47 (Napoli et al., 2024)..

### Scar classification

**Fig 2:**
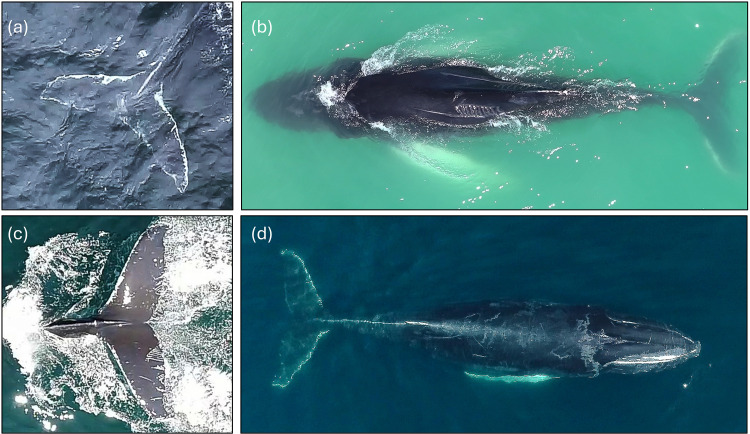
Examples of scar types assessed in the present study. **(a)** Entanglement scars, visible as white pigmented areas at the base and on the leading edge of the dorsal surface of the flukes; **(b)** Vessel strike scars, with propeller and skeg scars visible both on the left dorsal side anterior to the dorsal fin, and on the right dorsal side adjacent to the dorsal fin; **(c)** Killer whale rake marks visible on the trailing edge of the left dorsal side of the flukes; **(d)** Scarring on the right side of the jaw indicative of lateralized feeding behavior.

Observed scars were classified as follows (Fig 2):

*Entanglement scars* can be formed in body regions where gear causes lacerations on, or wraps around, a whale’s body. Entanglement scars on humpback whales appear white on dark pigmented areas or dark in white pigmented areas [27]. For example, after an entanglement, the leading edge of the fluke may appear white, rather than dark due to abrasions from gear wrapping around the fluke [28]. Gear entangled around another region of the whale’s body can trail off resulting in scrapes in this area or across the fluke [12,28]. In any body region, line-like scars that have parallel, mirrored, or criss-cross markings are indicative that there was something wrapped around or across the whale [21].

*Vessel Strike Scars.* Injuries sustained by vessel strikes can be divided into two categories; sharp or blunt trauma. Sharp trauma is caused by the propeller slicing into the whale and blunt trauma is caused by a collision with the hull. In the field it is difficult to detect a blunt trauma injury since it does not leave an easily detectable scar and its presence can only be confirmed during a necropsy [6,41]. Furthermore, blunt force injuries can be caused by other events and cannot always be definitively determined to be caused by vessels [30]. Sharp trauma injuries (propeller and/ or skeg scars) were the only scars categorized as vessel strike scars in this study. Vessel strikes involving the propeller can leave distinct, evenly spaced spiral shaped scars that occur when the propeller repeatedly cuts into the whale as it passes by [30,59]. In addition, the skeg on vessels can cause long, narrow scars on marine mammals in the direction of a boat’s travel [20,30,68,69].

*Killer whale scars* were identified by the distinct J shaped parallel and equidistant linear rake marks the teeth leave on the body of an individual [15,52].

*Jaw scuffing* was identified as an area of the rostrum that is scuffed from scraping along the bottom [16,17,47]. Humpback whales that show lateralization in feeding behavior will only have one side of their rostrum with the scar. Individuals showing scarring on one side of their rostrum, but not the other were considered to show lateralization [16,47].

**Fig 3:**
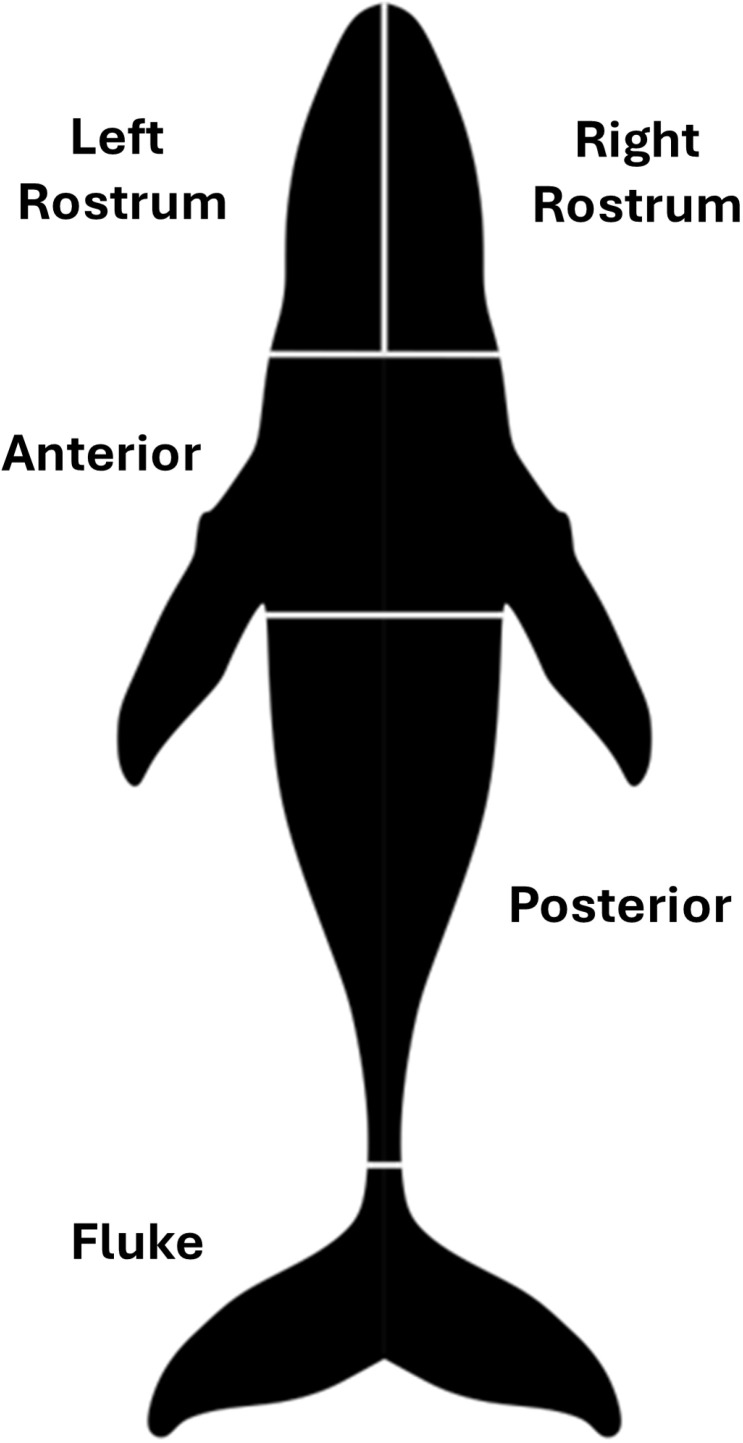
The body regions of humpback whales used to examine the location of scarring. Left and right rostrum were assessed separately to examine scarring reflective of handedness, while these two regions were assessed together when examining the general body region in which scars were observed.

Scarring types were confirmed by three experienced observers, and scars were only classified as vessel strike, entanglement, etc. when all three observers agreed. Initial characterizations of scars were verified by an expert with detailed knowledge of large whale scarring patterns. We only included scars that were deemed to be very likely or definitely due to entanglement, vessel strike, killer whale or jaw scuffing scars (e.g., Ramp et al. (2021)), respectively, and did not include marks or scars that were unlikely or uncertain to be from these sources. Scars that were observed on the sides of flukes or dorsal fins in aerial images were confirmed with boat-based photo-ID images whenever possible. The location of entanglement and vessel strike scars was assigned to one of four body regions, while scarring on the left and right rostrum reflective of jaw scuffing were assessed separately to examine lateralization (Fig 3). For each individual showing entanglement scarring, we assessed the number of points of entanglement as the number of regions that showed the occurrence of entanglement scarring.

### Analysis

We assessed differences between juveniles and adults in the presence of entanglement scars, vessel strike scars, killer whale scars, or handedness scarring (only one side showing jaw scuffing), respectively. Measurement error for individuals near the length threshold for adults and juveniles (11.47 m) could lead to misclassification, potentially affecting group comparisons [70]. To account for uncertainty in total length due to altitude error [64,70] and variation in total length among images of the same whale, we implemented a bootstrapping approach similar to that used in Napoli et al. (2024) [65] and Christiansen et al. (2018) [64]. For each whale, measurement errors associated with the specific drone model used were randomly sampled from the error distributions reported in the supplementary materials of Napoli et al. (2024). These errors accounting for both altitude and image variation were applied to the whale’s total length, and the age classification was reassigned accordingly. This process was repeated 1000 times, producing 1000 bootstrapped populations of humpback whales. For each bootstrapped population, we calculated summary statistics for the number of juveniles and adults with scars. Due to small sample sizes (values of less than 5 in any contingency table group), Fisher’s Exact tests were conducted on each population to assess differences in the presence of entanglement scars, vessel strike scars, killer whale scars, and handedness (only one side showing jaw scuffing) between juvenile and adult humpback whales [71]. A Chi-Square test was used to evaluate differences between juvenile and adults in jaw scuffing, as all contingency table values had larger sample sizes. Additionally to assess whether bottom feeding might be related to the occurrence of entanglement scarring, we conducted a Fisher’s Exact test in juveniles and adults, respectively, to compare the proportion of animals with and without jaw scuffing for whales showing entanglement scars. For all tests, reported *p*-values are indicative of values for the 1000 bootstrapped populations unless otherwise noted, with values summarized as greater than or less than a specified threshold where applicable.

## Results

Whole-body UAS imagery was obtained for 152 humpback whales in the New York Bight. A total of 30 individuals were excluded from the analysis since clear images were not obtained for the entire dorsal surface, or images were not of sufficient quality. Incorporating error in length measurements by bootstrapping 1000 populations resulted in a mean sample size of 77.4 juveniles (range: 76–79) and 44.6 adults (range: 43–46) for humpback whales. The vast majority of humpback whales assessed (107 of 122) showed evidence of entanglement or vessel strike scarring (86.8% overall; Table 2). The occurrence of entanglement scarring was similar between adults and juveniles (87.1% in adults vs. 86.8% in juveniles; mean p value = 0.95; all p > 0.782 in bootstrapped Fisher’s Exact test). For both adults and juveniles, entanglement scars were primarily observed on the flukes as well as the posterior region, and occurred less frequently on the rostrum and anterior regions of the body (Table 3). For individuals showing entanglement scars, the number of points of entanglement for each individual ranged from one to five, with the majority of individuals showing either one or two points of entanglement. Juveniles showed one point of entanglement most frequently, while adults showed two points of entanglement most frequently (Table 4). Evidence of scarring resulting from vessel strikes was observed in 11 juvenile whales (14.2%) and in one adult (2.2%). The p-value for the comparison of vessel strike scarring between adults and juveniles did not reach the threshold of statistical significance but suggested a trend (Fisher’s Exact test; mean *p*-value = 0.051, 139 of 1000 bootstrapped populations p < 0.05, 861 bootstrapped populations 0.05 < *p* < 0.054). Vessel strike scarring was observed most frequently in the posterior body region Table 3).

**Table 2:** Number of individual humpback whales with sufficient whole-body UAS imagery to quantify scarring, and the proportion of individuals showing different types of scarring for adults and juveniles, respectively. Values reflect the mean percentages from bootstrapping 1000 populations that incorporate error in length measurements. Left- and right-handed values reflect the proportion of individuals showing jaw scarring on only the left or right side, respectively.

Age Class	Mean Sample Size and Range	Entanglement	Vessel Strikes	Vessel Strike or Entanglement	Killer Whale	Jaw Scuffing	Left Handed	Right Handed	Handedness (either)
**Adult**	44.6	87.1%	2.2%	87.1%	4.4%	68.9%	15.9%	82.2%	98.2%
**Juvenile**	77.4	86.8%	14.2%	89.3%	11.6%	27.4%	22.1%	71.6%	93.6%
**All whales**	122	86.8%	9.8%	88.5%	9.0%	44.3%	18.5%	77.8%	96.3%

**Table 3:** Proportion of humpback whales showing entanglement, vessel strike and killer whale scars in different body regions. Values reflect the mean percentages from bootstrapping 1000 populations that incorporate error in length measurements (Mean adult n = 44.6, range = 43-46; mean juvenile n = 77.4, range = 76-79). Body regions are depicted in Fig 2.

	Rostrum	Anterior	Posterior	Fluke
**Entanglement Scarring**				
**Adult**	4.4%	6.7%	49.9%	84.8%
**Juvenile**	3.9%	7.8%	30.6%	83.3%
**All whales**	4.1%	7.4%	37.7%	83.6%
**Vessel Strike Scarring**				
**Adult**	0.0%	0.0%	2.2%	0.0%
**Juvenile**	1.3%	7.8%	10.3%	1.3%
**All whales**	0.8%	4.9%	7.4%	0.8%
**Killer whale Scarring**				
**Adult**	0.0%	0.0%	0.0%	4.4%
**Juvenile**	0.0%	0.0%	2.6%	9.0%
**All whales**	0.0%	0.0%	1.6%	7.3%

**Table 4:** The number of body regions with entanglement scars for humpback whales showing evidence of entanglement. Values reflect the mean percentages from bootstrapping 1000 populations that incorporate error in length measurements (Mean adult n = 38.9, range = 37-41; mean juvenile n = 67.1, range = 65-69).

Age class	One	Two	Three	Four	Five
**Adult**	37.5%	57.4%	5.1%	0.0%	0.0%
**Juvenile**	63.2%	32.3%	1.5%	0.0%	3.0%
**All whales**	53.8%	41.5%	2.8%	0.0%	1.9%

We observed jaw scuffing in 68.9% of adult humpback whales, and 27.4% of juveniles, representing significant differences between the age classes (mean *p*-value = 3.47 x 10^−5^, all *p* < 0.001, bootstrapped Chi-squared test). Scarring reflective of a right side bias (scarring on the right side of the jaw) was more common than that of the left side; a right side bias was observed in 77.8% of whales showing jaw scuffing vs. 18.5% for the left side. While a right bias was more common in both juveniles and adults, adults showed a somewhat stronger right bias, though this difference was not significant (Table 2; mean *p*-value = 0.46, all *p* > 0.194, bootstrapped Fisher’s Exact test). Adults showed somewhat more evidence of lateralization;in 98.2% of adults and 93.6% of juveniles showing jaw scuffing, the scuffing was restricted to one side of the jaw. This difference was not significant (mean *p*-value = 0.64, all *p *> 0.161, bootstrapped Fisher’s Exact test). Killer whale rake marks were observed more frequently in juveniles (11.6%) than adults (4.4%) (Table 2), though this difference was not significant (mean *p*-value = 0.26, all *p* > 0.204, bootstrapped Fisher’s Exact test).

Both juvenile and adult humpback whales with and without entanglement scars showed similar occurrence of jaw scuffing, reflecting bottom feeding (Table 5) (for juveniles, mean *p*-value = 0.94, all *p* > 0.721; for adults, mean *p*-value = 0.92, all *p* > 0.607, Fisher’s Exact test).

**Table 5:** Entanglement rates (proportion of individuals showing entanglement scarring) for adult and juvenile humpback whales, respectively, shown for whales with and without jaw scuffing. Values reflect the mean percentages from bootstrapping 1000 populations that incorporate error in length measurements.

Entanglement rates	With Jaw Scuffing	Without Jaw Scuffing
**All juveniles**	86.80%	
**Juveniles with entanglement**	85.8%	87.2%
**Juveniles without entanglement**	14.2%	12.8%
**Total**	100%	100%
**All adults**	87.10%	
**Adults with entanglement**	87.9%	85.0%
**Adults without entanglement**	12.1%	15.0%
**Total**	100%	100%

## Discussion

### Entanglement scars

The vast majority of humpback whales (86.8%) in the New York Bight showed scarring from entanglement in fishing gear. This finding is consistent with Ramp et al. (2021) who used boat-based peduncle and fluke images to examine scarring rates and found that 85% of humpback whales in the Gulf of St. Lawrence showed entanglement scarring. Our finding that 86.8% of juvenile humpback whales exhibit entanglement scarring suggests that humpbacks occurring in the New York Bight are frequently interacting with fishing gear even in their first years of life. Whales considered to be juveniles were less than 11.47 m in length (~8–11.47 m) and were therefore likely less than approximately five years of age [72], though recent length-at-age data are not available for Northwest Atlantic humpback whales. Robbins (2012) found that 16.9% of humpback whales in the Gulf of Maine acquired new entanglement scars each year (though many of these animals already showed evidence of scarring), and that there was a higher incidence of new and unhealed injuries in juveniles [13,27]. Our finding that adult humpback whales frequently showed more points of entanglement than juveniles suggests that through time, whales are interacting with fishing gear across multiple body regions. Future studies in the New York Bight region could assess changes to scars over time within the same individuals to assess the frequency of new entanglements. Additionally, continued monitoring of scarring patterns in humpback whales could allow temporal trends related to changes in anthropogenic threats to be assessed.

Our results suggest a higher rate of entanglement than that observed by Robbins (2012) in the Gulf of Maine (64.9%) using boat-based images. A comparison of UAS imagery and standard vessel-based photo-identification methods by Ramp et al. (2021) suggested that standard photo-ID methods, in comparison to whole-body UAS images, may underestimate entanglement rates in large whales as some regions of the body cannot be reliably observed. In the present study, entanglement scars were most frequently observed on the flukes. The posterior region, which includes the posterior caudal peduncle that is typically assessed during boat-based studies of scars, was also frequently affected by entanglement scarring. The occurrence of scarring in the posterior region observed in the present study for adults (49.9%) was comparable to that observed in prior studies on the posterior caudal peduncle in the Gulf of Maine (48–57%) [28]. Scars on the dorsal surfaces of humpback whales, including on the dorsal side of flukes, may not be visible in their entirety when using boat-based images. By providing imagery of the whole dorsal surface of an individual, UAS can be used to observe scarring in body regions that otherwise may only be fully assessed in deceased whales [12]. Furthermore, UAS can be used to provide images of multiple cetaceans at the same time, and can provide continuous imagery while the animals are surfacing [73]. In the present study, assessing scarring using a combination of video analysis and multiple screen grabs from the aerial footage of an individual was useful to confirm both the presence and absence of scars, particularly where factors such as glare or water disturbances create discontinuities in some (but not all) still images. UAS are also advantageous in cetacean studies in that they are minimally invasive, allowing images of cetaceans to be obtained for analysis with minimal disturbance [73–77].We observed few individuals with entanglement scars on the rostrum in this study (4.1% across all whales studied), while a study of entanglement that examined deceased whales found that 67% had entanglements in their mouth [21]. When reported entanglements included visible gear, it was most often observed on the flukes, followed by the mouth [18]. Rostrum entanglement can cause death through starvation or drowning from limited mobility [21]. Thus, animals with entanglements in this region may be less likely to survive than animals with entanglements in other body regions that may be able to withstand chronic entanglement, and consequently mouth entanglements may be less frequently detected in studies of live animals.

### Vessel strike scars

In the present study, 9.8% of all whales observed (juveniles and adults) had propeller or skeg scars. These results likely greatly underestimate the occurrence of vessel strikes as they reflect only whales that have survived vessel strikes and those with visible scars, as blunt force trauma cannot be detected using external images [30]. In comparison, 4.8% of whales observed in a prior study in the Gulf of Maine showed scars consistent with propeller or skeg scars [30]. This comparison suggests that humpbacks in the New York Bight may show higher rates of vessel strike than those in the Gulf of Maine. However, Hill et al. (2017) additionally categorized vessel strike scars as being due to other possible vessel strike injuries, defined as injuries that could not be assigned to other scarring categories but were unlikely to be social, foraging or entanglement injuries. The authors found that in total 14.7% of whales showed injuries consistent with vessel strike including this category of other potential injuries, which was not assessed in the present study.

We primarily observed vessel strike scars in juveniles, with 14.2% of juveniles and 2.2% of adults affected. The difference in the occurrence of vessel strike scars between juveniles and adults was or borderline statistical significance, likely due to our limited sample size along with the relatively infrequent occurrence of these scars in adults. A power analysis suggests that a sample size of at least 184 whales would be needed to observe a definitively significant difference (*p* < 0.05) given the relative numbers of adults and juveniles in our dataset and the prevalence of vessel strike scars. Further work could revisit this comparison as the sample size for this research grows in the future.

Vessel strike scars were most frequently observed in the anterior and posterior dorsal regions. These regions may be most vulnerable to vessel strike as the back is exposed while surfacing, and because humpbacks typically lift their tail stock before diving [30]. Our finding that juveniles in the New York Bight showed more evidence of vessel strike than adults is in contrast to Hill et al. (2017), who found that adult whales had a higher frequency of vessel strike scars and suggested that adult foraging in the upper water column may make them more vulnerable to vessel strike. In the New York Bight, juveniles are frequently observed surface feeding in shallow, inshore waters while adults primarily feed offshore [58–61]. The vast majority of individuals confirmed to have been killed by vessel strike in the New York Bight are juveniles and the shallow water surface feeding behavior of juvenile humpback whales in the New York Bight may put juveniles at a higher risk of vessel strike [57,61]. Our results, and the differences from Hill et al. (2017), reaffirm that juvenile humpbacks in the New York Bight may be particularly vulnerable to vessel strike and suggest that foraging behavior may play an important role in vulnerability to vessel strike. The differences between juveniles and adults in vessel strike, but not entanglement, scarring in our results suggest that vessel strikes are an important threat to juveniles in this region. Further, the observed differences from prior studies in the Gulf of Maine suggest that vessel strike scars may be obtained locally.

### Jaw scuffing and lateralization

We found that significantly more adults showed evidence of jaw scuffing than juveniles, although our dataset was dominated by juveniles. The marked difference in jaw scuffing observed between juveniles and adults suggests that adults are bottom feeding more often than juveniles. In addition, while the majority of humpback whales with jaw scuffing showed lateralization, a slightly, lower proportion of juveniles with jaw scuffing showed lateralization in comparison to adults. Both juveniles and adults showed a clear right-side bias, although this bias was less pronounced in juveniles. Studies of humpback whales in the southern Gulf of Maine have found a strong right-side bias in lateralized foraging behavior [16,47]. Many other marine mammals exhibit lateralized foraging behavior [78] and have a right-side bias as in humans [47,79]. Humpbacks learn and adapt foraging skills over time, and one to three years of age may be a critical period for learning feeding behavior [80]. Tagging studies have suggested that lateralized feeding behavior in humpback whales increases with age, perhaps because juveniles take more time to hone their foraging skills [47]. Our finding that adults show more jaw scuffing than juveniles further highlights age-specific differences in habitat use and foraging behavior, which may have implications for exposure to anthropogenic threats [61].

In North Atlantic right whale, interactions with the seafloor have been used to reflect risk of entanglement in fishing gear associated with the bottom [48]. Our results suggest that the majority of adult humpback whales bottom feed regularly, and prior studies have suggested that bottom feeding in regions of high bottom-fishing effort could present a high entanglement risk [44]. We did not observe a link between bottom feeding and risk of entanglement in humpback whales, as humpback whales with and without jaw scuffing showed similar levels of entanglement scarring, though jaw scuffing provides a coarse indicator of bottom feeding.

### Killer whale scars

The proportion of whales showing killer whale rake marks varies considerably between regions [15,81], and the prevalence of killer whale scars observed across both adult and juvenile humpback whales in the New York Bight (9.0%) was similar to that reported for humpback whales in the Gulf of Maine (9.3%) [15]. Killer whales are thought to primarily target young animals [50,52,81], and we found that a somewhat greater proportion of juvenile humpback whales (11.6%) than adults (4.4%) had killer whale rake marks, although this difference was not significant. As for vessel strike scars, a power analysis suggests that a bigger sample size (470 individuals) would be needed to demonstrate a significant difference between adults and juveniles given the relative occurrence of these age classes and of killer whale scars. Most killer whale scars were observed on the flukes, as well as on the posterior region, which is consistent with prior observations as rorquals typically flee in response to killer whale interactions [14,82]. Despite co-occurring in breeding grounds, humpback whales show substantial differences in killer whale scarring across the Northwest Atlantic foraging grounds, suggesting that most killer whale attacks occur during migration or in foraging grounds [15,81]. In both gray whales and humpback whales, mother-calf pairs preferentially use nearshore waters during migrations, and killer whale predation in offshore waters is thought to be a driver of this inshore habitat use [83]. Juvenile humpback whales occur in inshore waters in the New York Bight, and are typically solitary, while both juveniles and adults occur offshore and feed cooperatively in offshore waters (Stepanuk et al. 2021). It is possible that avoidance of killer whales in offshore waters by unaccompanied juvenile humpback whales could be a driver of their inshore habitat use, which may in turn put juvenile whales at higher risk of vessel strike. While killer whale sightings in this region are infrequent, they do occur periodically; for example, a group of 4 killer whales was observed in the region in June 2023 during aerial surveys (Pers. comm., O. O’Brien).

## Conclusions

Our results highlight the extent to which humpback whales occurring in the New York Bight are impacted by multiple anthropogenic threats even in the first years of their life. Our analysis of UAS imagery to assess scarring in humpback whales suggests that this approach can be particularly useful for assessing anthropogenic scars, since scarring was frequently observed in dorsal regions which may be difficult to capture from boat-based photographs. Further, whole-body UAS imagery allowed multiple scar types to be assessed simultaneously. The New York Bight has only been used regularly by humpback whales as a foraging area since approximately 2011 [59,61,84,85], and until recently there has been limited information on the detailed habitat use of large whales in this region [86]. Since New York waters have been a hotspot of humpback whale strandings and vessel strikes during the UME [57], there is a great deal of interest in understanding anthropogenic threats in this region. Our results support prior suggestions that juvenile whiles may be particularly vulnerable to vessel strike in the New York Bight, and provide further evidence for age-specific differences in habitat use and foraging behavior in humpback whales. Continued studies of humpback whales in the New York Bight will improve our understanding of anthropogenic impacts on juvenile whales, and the implications of behavioral differences between juvenile and adult whales for the risk of anthropogenic threats.

## Supporting information

S1 File Table summarizing scars identified for each individual humpback whale and body region, along with the year of observation, total length and age class of each individual, drone model used to obtain images, and altitude method used to assess total length. Abbreviations are as follows: L, left; R, right; ES, entanglement scar; VS, vessel strike scar; KW, killer whale scar.
(XLSX)
